# Mapping Natural Sugars Metabolism in Acute Myeloid Leukaemia Using 2D Nuclear Magnetic Resonance Spectroscopy

**DOI:** 10.3390/cancers16213576

**Published:** 2024-10-23

**Authors:** Christina Muhs, Islam Alshamleh, Christian Richter, Hubert Serve, Harald Schwalbe

**Affiliations:** 1 Center for Biomolecular Magnetic Resonance (BMRZ), Institute for Organic Chemistry and Chemical Biology, Goethe University, 60438 Frankfurt am Main, Germany; muhs@nmr.uni-frankfurt.de (C.M.); islam.alshamleh@cruk.cam.ac.uk (I.A.); ric@nmr.uni-frankfurt.de (C.R.); 2 German Cancer Consortium (DKTK), Partner Site Frankfurt/Mainz, 60528 Frankfurt am Main, Germany; serve@em.uni-frankfurt.de; 3 University Cancer Center (UCT) Frankfurt, University Hospital, Goethe University, 60590 Frankfurt am Main, Germany; 4 Frankfurt Cancer Institute, Goethe University, 60596 Frankfurt am Main, Germany

**Keywords:** AML, sugar metabolism, isotope tracing, NMR spectroscopy

## Abstract

Metabolism plays a central role in cancer progression. Rewiring glucose metabolism is essential for fulfilling the high energy and biosynthetic demands as well as for the development of drug resistance. Nevertheless, the role of other diet-abundant natural sugars is not fully understood. In this study, we performed a comprehensive 2D NMR spectroscopy tracer-based assay with a panel of ^13^C-labelled sugars (glucose, fructose, galactose, mannose and xylose). We assigned over 100 NMR signals from metabolites derived from each sugar and mapped them to metabolic pathways, uncovering two novel findings. First, we demonstrated that mannose has a semi-identical metabolic profile to that of glucose with similar label incorporation patterns. Second, next to the known role of fructose in driving one-carbon metabolism, we explained the equally important contribution of galactose to this pathway. Interestingly, we demonstrated that cells growing with either fructose or galactose became less sensitive to certain one-carbon metabolism inhibitors such as 5-Flurouracil and SHIN1. In summary, this study presents the differential metabolism of natural sugars, demonstrating that mannose has a comparable profile to that of glucose. Conversely, galactose and fructose contribute to a greater extent to one-carbon metabolism, which makes them important modulators for inhibitors targeting this pathway. To our knowledge, this is the first NMR study to comprehensively investigate the metabolism of key natural sugars in AML and cancer.

## Simple Summary

The rapid growth of cancer cells is fuelled by excessive sugar uptake and utilisation. While laboratory experiments modelling cancer cell metabolism primarily focus on glucose, in this paper, we address the unexplored roles of other sugars (fructose, galactose, mannose and xylose) that are heavily abundant in the diet. In this study, we feed cancer cells with stable isotopes of those sugars and, using nuclear magnetic resonance spectroscopy, we assess how they are being metabolised by acute myeloid leukaemia cells. We provide a metabolic map detailing the unique metabolism of each of those sugars in cancer cells and we identify a novel role of galactose in supporting their building blocks’ biosynthesis. We also demonstrate the ability of galactose to modulate cancer cells’ responses to various drugs and chemotherapies. This study highlights the importance of mimicking human dietary compositions when studying cancer cell metabolism in the lab.

## 1. Introduction

Cancer metabolism is dysregulated and reprogrammed to fulfil the high energy demands and synthetic needs of cancer cells [1,2,3]. Altered glucose metabolism has been well studied in cancer cells, which convert their glucose to lactate regardless of oxygen levels, a phenomenon known as the Warburg effect [4,5]. These findings led to several preclinical and clinical studies testing new therapies against glucose metabolism [6,7]. Nevertheless, many of those studies did not yield satisfactory clinical results, in part due to the metabolic adaptation of the cancer cells and their ability to utilise alternative nutrients [8].

While glucose is the main sugar used in energy generation, human dietary intake entails several other sugars, including fructose, galactose, xylose, mannose and sucrose [9,10,11]. Several studies have addressed the role of natural sugars in cancer, such as fructose [12,13] and mannose [14,15] in acute myeloid leukaemia (AML). In fact, AML cells were shown to upregulate fructose transporters to enhance their one-carbon metabolism and supply for nucleotide synthesis. Nevertheless, the metabolism of most natural sugars has yet to be fully explored. Understanding these metabolic drivers is particularly important in cancer considering the high adaptability of cancer cells to stress conditions and nutrient scarcity.

In this study, we present a comprehensive NMR-based metabolic map of natural sugars in AML using tracer-based assays. We demonstrate the ability of certain sugars to fully substitute glucose, and we determine novel roles of other sugars in supplementing certain metabolic pathways and modulating drug responses.

## 2. Materials and Methods

### 2.1. Cell Culture and Maintenance

Molm13, HEL, MV4-11, THP1 and Kasumi cell lines were obtained from the DSMZ (Deutsche Sammlung von Mikroorganismen und Zellkultur GmbH, Braunschweig, Germany) and were maintained at 37 °C with 5% CO_2_. RPMI 1640 (Gibco/ThermoFisher, Waltham, MA, USA) standard medium was supplemented with 10% heat-inactivated foetal bovine serum (FBS) (Sigma Aldrich, Burlington, MA, USA) and 1% P/S (Penicillin-Streptomycin 10.000 U/mL). Cells were grown for at least 14 days before the experiments were performed. Glucose-free RPMI 1640 medium (Gibco) was supplemented with 10% heat-inactivated FBS (foetal bovine serum from Sigma Aldrich) and 1% PS (Penicillin-Streptomycin 10.000 U/mL) as well as 10 mM of the corresponding sugar (glucose, fructose, mannose, galactose, xylose and sucrose obtained from Sigma Aldrich). The cell density was adjusted to between 5 and 8 × 10^5^ cell/mL. The cells were split every two to three days and supplied with fresh medium. No ethics approval was needed for the cell lines experiment (no primary patient materials were used, and no animal experiments were performed).

### 2.2. Proliferation Assays

AML cell lines were seeded at a density of 4 × 10^5^ cell/mL and grown in the presence of the different sugars (unlabelled). Cells were counted every two days and replated back to the original density in fresh medium for a period of eight days. Cells were counted using Trypan blue on a Countness 3 cell counter (Invitrogen).

### 2.3. Cell Viability Assays

After cells were grown with the corresponding sugar for a given time, an equal number of cells were plated in 96-well plates (Greiner, Kremsmünster, Austria, (chimney 96-well white plates)). Next, 20 μL Cell Titer-Glo (Promega, Madison, Wisconsin, USA) was added to the cells, briefly mixed then incubated at 37 °C for 10 min before reading luminescence with a Tecan Reader (Infinite M200 Pro, TECAN, Männedorf, Switzerland).

### 2.4. Consumption Rates

To measure sugar consumption, AML cells were grown with unlabelled sugars at a density of 8 × 10^5^ cells/mL. Twenty-four hours later, media samples were harvested and analysed by NMR as described in the NMR section below.

### 2.5. ^13^C Tracer-Based Assay

Glucose-free media (RPMI 1640 from Gibco) were supplemented with 10 mM ^13^C-labelled sugars (mannose, galactose, xylose, fructose, and glucose) (obtained from Eurisotop), 10% heat-inactivated FBS and 1% PS. Cells were washed then resuspended with the corresponding media at a density of around 8 × 10^5^ cell/mL (an approximate total of 40 million cells by the day of extraction). Cells were grown at 37 °C and 5% CO_2_. Twenty-four hours later, cells were extracted as described below.

### 2.6. Metabolites Extraction

On the day of extraction (24 h post-labelling), cells were counted, and 40 million cells were centrifugated at 2000 rpm for 4 min. After discarding the supernatant, the pellet was washed with 1 mL PBS and centrifugated again at 13.000 rpm for 20 s and 4 °C. The supernatant was discarded, and the pellet was lysed with 400 μL methanol and vortexed for 10 s. Samples were then transferred to glass vials and 375 μL distilled water and 400 μL chloroform were added. The samples were vortexed for 40 s, then placed on ice for 10 min. The samples were then centrifugated at 4000 rpm for 10 min at 4 °C in a swingout rotor and samples were placed at room temperature for 10 minutes. The upper polar phase was obtained using a Hamilton syringe and dried in a speed vac at 4 °C.

### 2.7. Sample Preparation and NMR Measurements

Dried metabolites extracts were resuspended in sodium phosphate buffer (pH 7, 100% D_2_O and 0.5 mM TMSP. Samples were loaded in the NMR tubes (either 40 µL in 1.7 mm or 190 µL in 3 mm tubes, Bruker LabScape Scream, Billerica, MA, USA). For media samples (consumption assays), 190 µL media were transferred into 3mm NMR tubes. Samples were measured on a 600MHz NEO Bruker spectrometer equipped with a Cryo TCI probe at 298 K. We recorded Spectra with 57,142 time-domain points, 512 scans using the noesygppr1d pulse programme. ^1^H-^13^C- Heteronuclear Single Quantum Coherence (HSQC) spectra were recorded using the phase sensitive standard Bruker pulse programme hsqcctetgpsp with 1426 complex points in the direct dimension and 1328 increments in the indirect dimension, 16–64 scans and 50% NUS (non-uniform sampling) at 298 K. For the purpose of validating the chemical shift assignments, HMQC-COSY spectra were recorded using the standard Bruker pulse programme h2bcetgpl3 with 2048 complex points in the direct dimension, 256 increments in the indirect dimension and 128 scans. Spectral assignment was performed with the help of online available metabolites reference spectra from the Human Metabolome Data Base (HMDB) and the Biological Magnetic Resonance Bank (BMRB). All spectra were processed in a Bruker Topspin. The 1D Spectra were analysed using Mestrenova and the ^1^H-^13^C-HSQC-Spectra were analysed using nmrfam Sparky 1.470.

### 2.8. Drug Treatment

AML cell lines were grown with the different sugars (unlabelled) for 6 days to adapt them prior to drug treatments. Cells were then seeded at a density of 2.2 × 10^5^ cell/mL in 96-well plates with the corresponding sugars and treated with different concentrations of the inhibitors (5-Flurouracil and SHIN1 (obtained from Selleckchem, Houston, TA, USA). Cell viability was recorded 72 h later using Cell Titer-Glo as described above. The data analysis and statistical analysis were performed in GraphPad Prism 10.3.1. The experiment was performed in technical triplicates and repeated twice.

## 3. Results

### 3.1. AML Cells Grow Differentially When Grown with Different Natural Sugars

**Figure 1:**
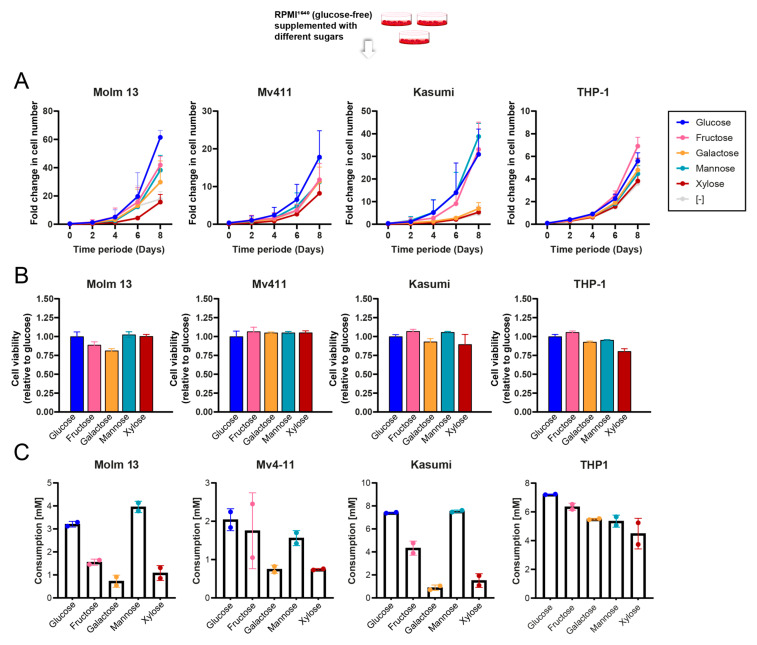
Proliferation and metabolic adaptability of AML cells to natural sugars. (**A**) Comparisons of growth rates of 4 AML cell lines growing with different sugars over a period of 8 days. (**B**) Viability of 4 different AML cell lines on day 8 after growing in different sugars relative to day 0. (**C**) Sugar consumption in 4 different AML cell lines after 24 h of sugar switch (*n* = 2–3 technical replicates).

We started by assessing the ability of AML cells to survive on different sugar sources. We grew four AML cell lines (Molm13, Kasumi, MV4-11 and THP1) over a period of 8 days in the presence of either glucose, mannose, fructose, galactose or xylose. Cell lines varied in their ability to grow in the absence of glucose. Generally, most cell lines could grow with mannose and fructose at a comparable rate to that of glucose (Figure 1A). However, when growing under galactose-rich media, the proliferation rates were slower for most cell lines, which is in line with the associated slow metabolism rate of galactose [16]. Finally, cells grown with xylose had the slowest growth pattern observed across all cell lines and cells took longer to adapt and grow after the sugar switch. Nevertheless, when we looked at the ATP levels in the cells eight days after sugar switch, albeit with slight cell line variations, there were only minor differences in ATP content per cell under different sugars (Figure 1B).

Next, we assessed the consumption rates for each of those sugars within 24 h of the sugar switch. This was in line with the proliferation patterns for most cell lines, whereby cells consumed similar amounts of glucose and mannose and slightly less fructose, galactose and xylose (Figure 1C).

### 3.2. Mapping Sugar Metabolism by 2D NMR Spectroscopy

**Figure 2:**
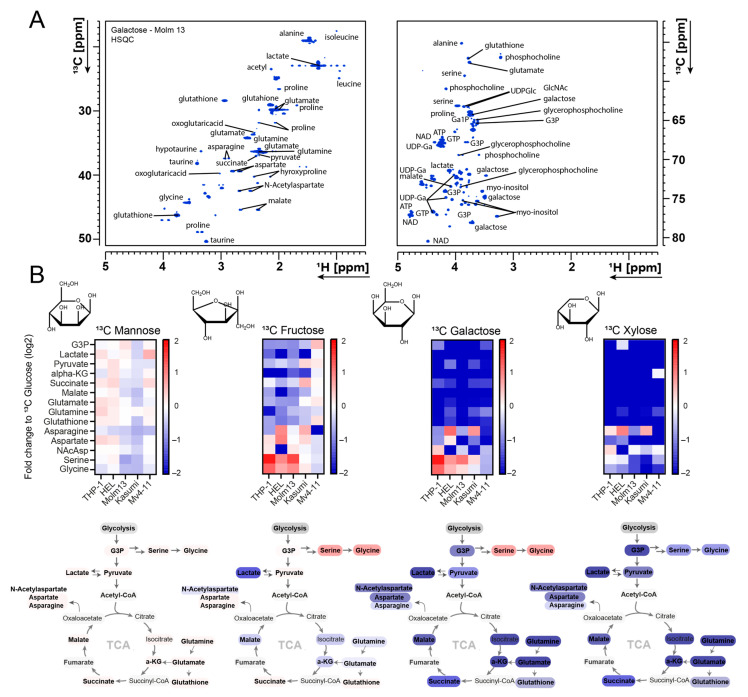
NMR metabolic profiling of natural sugars metabolism in AML. (**A**) ^1^H-^13^C 2D-HSQC example NMR spectrum of ^13^C galactose labelling and spectral assignment in Molm13 cells. Full ^1^H-^13^C 2D-HSQC of all sugars for Molm 13 is shown in Supplementary Figure S4. (**B**) Heat maps displaying fold change in ^13^C intensity of selected metabolites labelled with different ^13^C sugars (relative to glucose) in 5 AML cell lines. Full heat map is shown in Supplementary Figure S1. G3P (glycerol-3-phosphate), Ga1P (galactose-1-phosphate), UDP-Glc (UDP-glucose), alpha-KG (alpha-ketoglutaric acid), NAcAsp (N-acetyl aspartate), ATP (Adenosine triphosphate), NAD (nicotinamide adenine dinucleotide), UDP-Ga (uridine diphosphate galactose), GTP (guanosine triphosphate).

Next, we aimed at assessing how leukaemic cells metabolise different sugars and whether these sugars are substitutable or have different metabolic utilisations. We performed an NMR tracer-based assay using different ^13^C-labelled sugars in five AML cell lines. Cells were fed with the correspondent ^13^C-labelled sugar for 24 h, then metabolites were extracted and analysed by NMR spectroscopy. The analysis of the two-dimensional ^1^H-^13^C-HSQC spectra using an untargeted approach enabled the assignment and quantification of over 100 signals per spectrum. Figure 2A shows a sample assigned spectrum from Molm13 cells that were fed with ^13^C galactose.

Interestingly, mannose-fed cells had an almost identical label incorporation profile to that of glucose with very minor differences between the cell lines (Figure 2B). For instance, we observed similar ^13^C label incorporation rates into lactate, glutamate, phosphocholine, serine, glutathione and numerous other metabolites.

Overall, ^13^C intensities arising from other sugars were slightly lower, in line with the slower proliferation patterns observed with the corresponding sugars (fructose flowed by galactose then xylose). We observed relatively low label incorporation from xylose into downstream metabolites compared to other sugars. We could not detect any strong label enrichment pattern in any downstream metabolic pathway in any of the cell lines. While ^13^C intensities in certain metabolites, such as taurine, myo-inositol and phosphocholine, were comparable to those observed in ^13^C glucose samples, they were most likely not derived from ^13^C xylose, and instead were derived from other sources. This is in line with the low proliferative capacity of cells when grown with xylose. Therefore, we did not follow up with xylose and instead focused on fructose and galactose.

**Figure 3:**
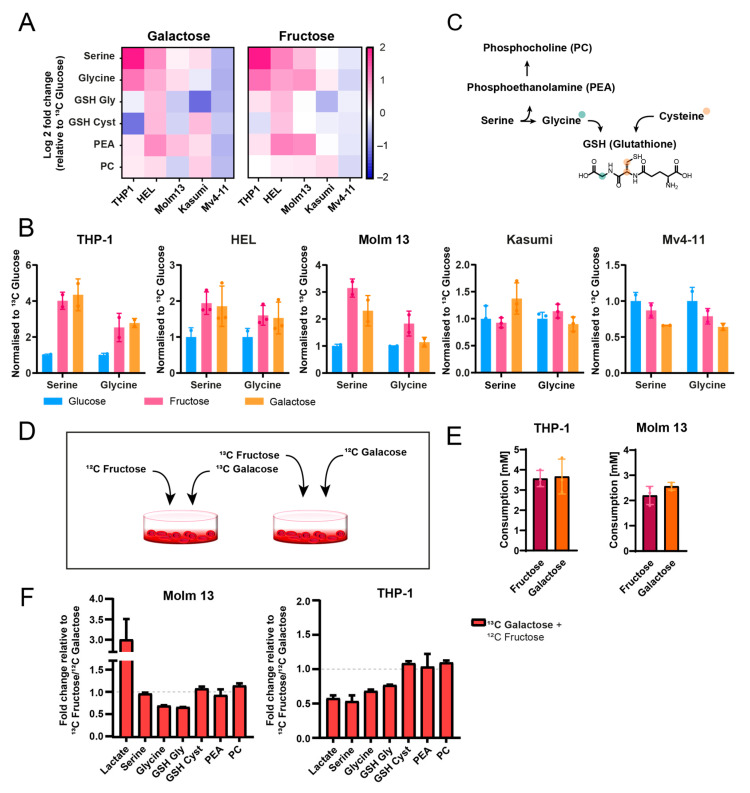
Galactose and fructose support one-carbon metabolism. (**A**) Heat map of the fold change of ^13^C label incorporation into a selection of metabolites depicting one-carbon and redox metabolism. (**B**) Overview of fructose and galactose metabolic fates based on the ^13^C label incorporation patterns. (**C**) ^13^C label incorporation ratio from fructose or galactose into serine and glycine in 5 different AML cell lines. (**D**) Schematic illustration of the two different settings for the contrast; (i) ^12^C galactose + ^13^C fructose or (ii) ^13^C galactose + ^12^C fructose. (**E**) Fructose and galactose consumption in a contrasting metabolic assay. (**F**) Fold change of ^13^C label enrichment from the contrasting metabolic assay of galactose and fructose. Full bar plot is shown in Supplementary Figure S3.

Interestingly, fructose and galactose exhibited similar labelling patterns (except for labelling in their direct metabolic derivatives (i.e., Ga1P and UDP-galactose) but their patterns differed from those of glucose. They both led to strong ^13^C label enrichment in serine and glycine (Figure 3A,B). While this has been reported previously for fructose [12], we explain here the metabolic utilisation of galactose for feeding the one-carbon metabolism. We only observed this enrichment in Molm13, THP1 and HEL cells, but not in MV4-11 and Kasumi cells, and this was consistent for both fructose and galactose. Interestingly, the fate of this enrichment in glycine and serine varied between the cell lines, whereby HEL cells had a fraction of this label enrichment in serine/glycine transferred to glutathione (originating from serine-derived cysteine, which could not be quantified due to sensitivity limitations), and to phosphocholine synthesis (the latter was also observed for THP1 cells). Nonetheless, Molm13 cells did not show any of this label transfer, suggesting stronger shuttling of the ^13^C label from serine and glycine towards nucleotide biosynthesis.

Finally, for the cell lines that did not show this pattern (Mv4-11 and Kasumi), we did not see an alternative label incorporation fate, suggesting cell line-specific utilisation of fructose and galactose, which might be associated with certain genetic backgrounds. We evaluated whether we could explain this by assessing the expression levels of enzymes and transporters involved in galactose metabolism in publicly available gene expression data [17]. While we did not see differences between those cell lines in their *GALK1* or *GALM* (both mediate galactose incorporation into glycolysis), we saw 4–5-fold higher expression levels of *GLUT14* (galactose transporter) in cells that showed galactose enrichment in the one-carbon metabolism (Molm13, HEL and THP1) compared to cell lines that did not (MV4-11 and Kasumi (Supplementary Figure S2)).

Given that these data suggest that galactose might be playing an equally important role in providing for one-carbon metabolism compared to fructose, we sought to further validate this by comparing the relative contribution of both sugars to one-carbon metabolism in a competitive metabolic assay. We grew Molm13 and THP1 cells in a 1:1 mixture of galactose and fructose (in the absence of glucose) in two different settings; (i) ^12^C galactose + ^13^C fructose or (ii) ^13^C galactose + ^12^C fructose. Interestingly, while both sugars were taken up at similar rates (Figure 3E), there were some differences between the cell lines, with THP1 exhibiting comparable label incorporation from both sugars into serine and glycine (although slightly lower incorporation from galactose) (Figure 3F). Molm13 had higher enrichment from fructose vs. glucose in serine and glycine. With the exception of significantly higher labelling in lactate in Molm13 (a 3-fold increase), labelling did not differ between the two sugars in glutathione, phosphoethanolamine or phosphocholine.

### 3.3. Galactose Reduces Sensitivity to One-Carbon Metabolism Inhibition

**Figure 4:**
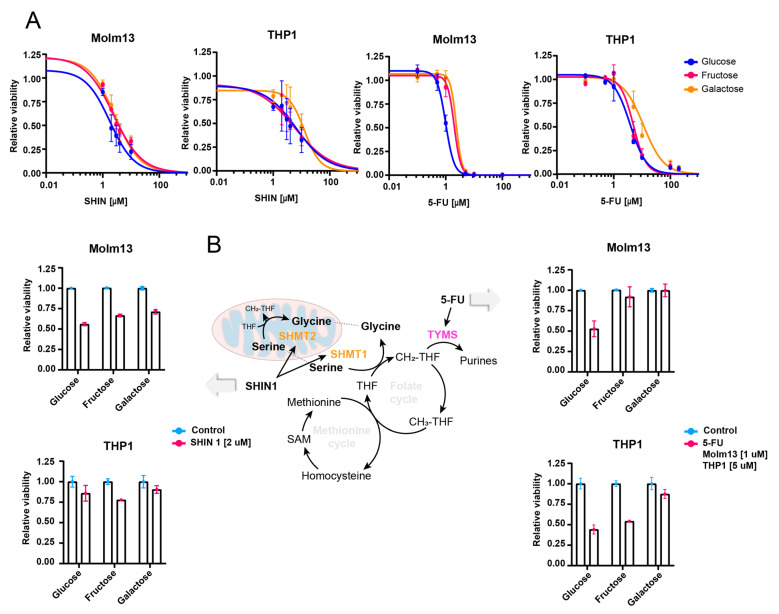
Galactose reduces sensitivity to one-carbon metabolism inhibitors. (**A**) IC50 curves of Molm13 and THP1 cells treated with SHIN1 and 5-FU. Cells were previously adapted for 6 days’ growth in media containing either glucose, fructose or galactose. Relative survival of Molm13 and THP1 treated with 5-FU or SHIN1 at approximately IC50 concentrations. (**B**) Schematic displaying the one-carbon metabolism inhibitors [19,20].

Finally, given the strong shuttling of galactose towards one-carbon metabolism, we aimed at assessing whether cells growing with galactose/fructose would be more resistant to inhibitors targeting the one-carbon metabolism such as 5-Flurouracil (5-FU) and the SHMT1/2 inhibitor SHIN1 [18]. First, we adapted Molm13 and THP1 cells to growing with either glucose, galactose or fructose for 6 days before treatment. Interestingly, we observed lower sensitivity to both inhibitors in cells growing with galactose and fructose compared to those growing with glucose (Figure 4A,B). The pattern was stronger in Molm13 compared to THP1 cells. Although the effect size was not very large, these data further confirm our findings on the role of galactose and fructose in driving the one-carbon metabolism and hence, desensitising cancer cells to inhibitors of this pathway.

## 4. Discussion

Glucose metabolism has been well studied in cancer. Although some studies have explored fructose [12] and mannose metabolism [14,15], a comprehensive understanding of natural sugars is still lacking. In this study, we performed an NMR tracer-based metabolic analysis and mapped the metabolic fates of most abundant dietary sugars in AML cell lines. To our knowledge, this is the first NMR (or mass spectrometry) study that provides an overall picture of sugars metabolism in cancer.

Our untargeted NMR approach is significantly more comprehensive than most published NMR metabolic screenings in terms of spectral assignment and the number of signals quantified. We achieved a spectral assignment of over 72%. Given the significantly higher reproducibility of NMR compared to mass spectrometry and its non-destructive nature, our study gives a strong boost to NMR spectroscopy [21,22,23] in steady-state stable isotope tracing.

We highlight two main findings in this study: first, the role of galactose (next to fructose) in driving the one-carbon metabolism in the absence of glucose. One-carbon metabolism plays a pivotal role in nucleotide synthesis, methylation, and redox balance, and this is particularly important in cancer cells given their high biosynthetic and redox control demands [24]. Previously, fructose has been reported to be essential for feeding the one-carbon metabolism at a rate that is higher than that of glucose [12]. Here, we reveal an equally important role of galactose in supplying the one-carbon metabolism. We also show in a competitive metabolic assay between galactose and fructose that AML cells comparably rely on and consume galactose to supply one-carbon metabolism, albeit at slightly lower rates in some cell lines. This is presumably due to the slow metabolism of galactose [16], which, in contrast to glucose, does not yield any net ATP when metabolised through glycolysis. This slower metabolism of galactose is also reflected in the slower proliferation of cells grown with galactose. Given all of this (and in spite of the high label incorporation observed in lactate), galactose is unlikely to be essential for energy generation but rather for nucleotide synthesis. This is illustrated in its desensitisation effect to one-carbon metabolism inhibitors. While we did not assess its importance beyond that, labelling in glutathione suggests the importance of galactose for redox metabolites [25] and labelling in phosphocholine suggests a role in lipid metabolism [26].

It is worthy of note that this role of galactose in supplying the one-carbon metabolism is not observed in all AML genetic backgrounds. In fact, our analysis of publicly available gene expression data suggests that this only happens in cell lines with higher expression of the galactose transporter GLUT-14. Indeed, a newly emerging report has demonstrated a correlation between poor disease-free survival and galactose metabolism in breast cancer patients [27]. Moreover, the expression level of GALK1 (the enzyme that converts galactose to galactose-1-phosphate) was shown to correlate with poor prognosis in glioblastoma [28] as well as in colorectal cancer [29].

Importantly, we show in this study that a galactose-rich medium reduces sensitivity to 5-flurouracil and SHMT1 inhibition. This is highly relevant when studying drug sensitivities in vitro where cells are grown in media that lack many molecules that are present in a physiological context (including galactose, which is amongst the most abundant dietary sugars (monomer of lactose)). An example of this is the role of uric acid in driving resistance towards 5-flurouracil, which was only observed when Cantor et al. designed a physiological cell culture that contains uric acid [30].

The second finding of this study was the ability of mannose to substitute glucose as a source for glycolysis and most other metabolic pathways. This potential for substitution was observed not only in metabolic labelling patterns but also in sugar consumption rates, ATP levels and proliferation ability. These results are in line with a recent report on mannose metabolism and its role in driving glycolysis in the absence of glucose [14]. Another report [15] revealed a slightly different role of mannose in supplying fatty acid metabolism via the high expression of the mannose-6-phosphate isomerase (MPI). Inhibition of MPI increased AML cells’ susceptibility to FLT3-inhibitors by increasing ferroptosis. While our study does not address fatty acid metabolism (only polar metabolites were analysed), our data do not contradict that report, given the role of glucose (and presumably mannose) in feeding multiple metabolic pathways, including fatty acid metabolism.

Furthermore, we did not observe any particular labelling pattern for xylose in any downstream metabolic network, which, together with its insufficiency for driving cell proliferation, suggests its insignificance in supporting metabolic networks.

Finally, our results need to be further validated in vivo due to the limitation of cell culture models, particularly in the context of metabolism (the lack of complete physiological nutrient composition and its implications for drug responses and metabolic adaptions).

## 5. Conclusions

In summary, our study is the first comprehensive NMR metabolic study that maps the metabolism of natural sugars in AML. Our untargeted approach and the nearly full spectral assignment provided useful insights into the differential metabolism of those sugars. We have revealed an essential role of galactose in feeding the one-carbon metabolism and demonstrated its implications for one-carbon metabolism inhibitors. Further work is needed to investigate less abundant dietary sugars and also to explore lipid metabolism.

## Data Availability

All supplementary data generated for this work are available online with this manuscript.

## Supplementary Materials

The following supporting information can be downloaded at https://www.mdpi.com/article/10.3390/cancers16213576/s1. Supplementary Figure S1. Full heatmap showing the fold change of ^13^C intensity (relative to ^13^C glucose) in a large panel of metabolites labelled with different ^13^C sugars in 5 AML cell lines. Supplementary Figure S2. Expression levels of galactose metabolism-related genes. Gene expression data for enzymes and nutrient transporters involved in galactose metabolism (GALM, GALK1, GLUT3 and GLUT14) in 5 AML cell lines. Supplementary Figure S3. Metabolic competition assay of fructose and galactose. Bar plots displaying the ^13^C intensity of a panel of metabolites labelled with ^13^C galactose (mixed 1:1 with ^12^C Fructose) in Molm13 and THP1 cells relative to those labelled with ^13^C fructose (mixed 1:1 with ^12^C Galactose. Supplementary Figure S4. ^1^H-^13^C 2D-HSQC NMR spectra of ^13^C labelled sugars (glucose, fructose, mannose, galactose and xylose. An overlaid spectrum of all sugars is also shown.
