# Supplementary material for: Mapping Natural Sugars Metabolism in Acute Myeloid Leukaemia Using 2D Nuclear Magnetic Resonance Spectroscopy
Source: Cancers (Basel). 2024 Oct 23;16(21):3576. doi: 10.3390/cancers16213576 (PMC11545164; doi:10.3390/cancers16213576)
Supplement: Supplementary file 1 [file cancers-16-03576-s001.zip › cancers-3242911-supplementary.pdf]

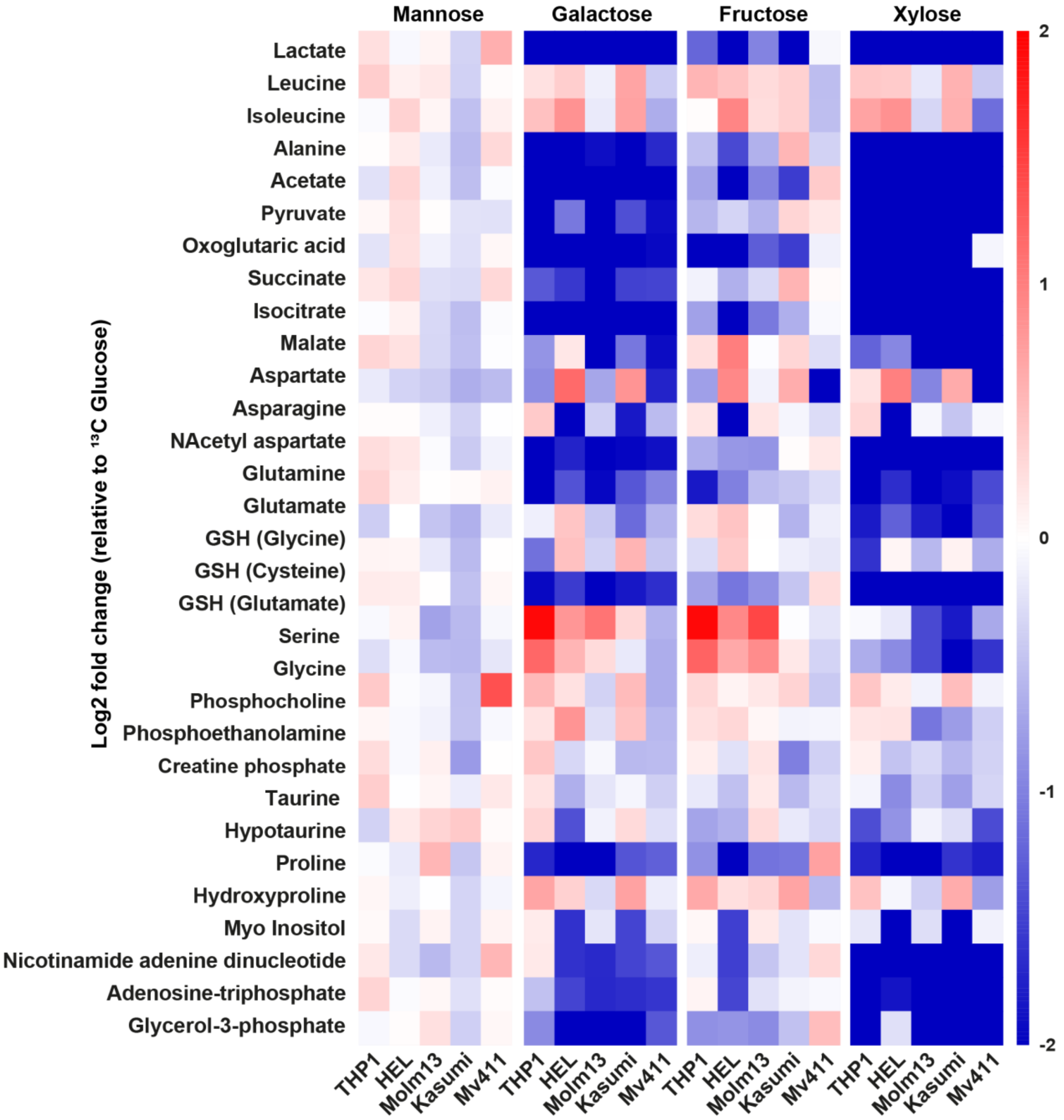

Supplementary Figure S1. Full heatmap showing the fold change of ^{13}C intensity (relative to ^{13}C glucose) in a large panel of metabolites labelled with different ^{13}C sugars in 5 AML cell lines.

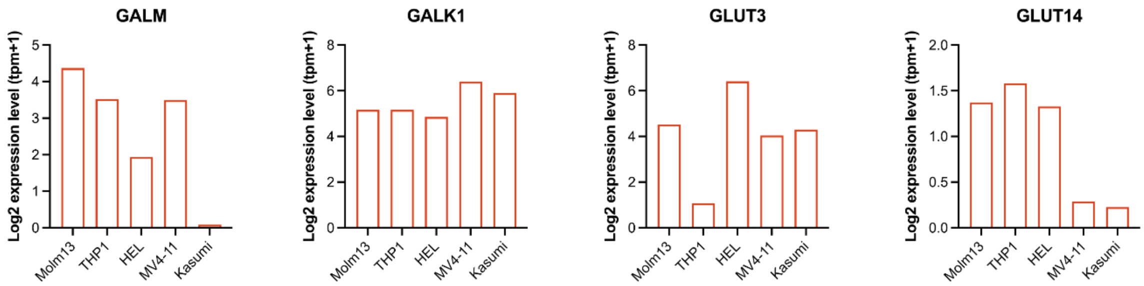

Supplementary Figure S2. Expression levels of galactose metabolism-related genes. Gene expression data for enzymes and nutrient transporters involved in galactose metabolism (GALM, GALK1, GLUT3 and GLUT14) in 5 AML cell lines.

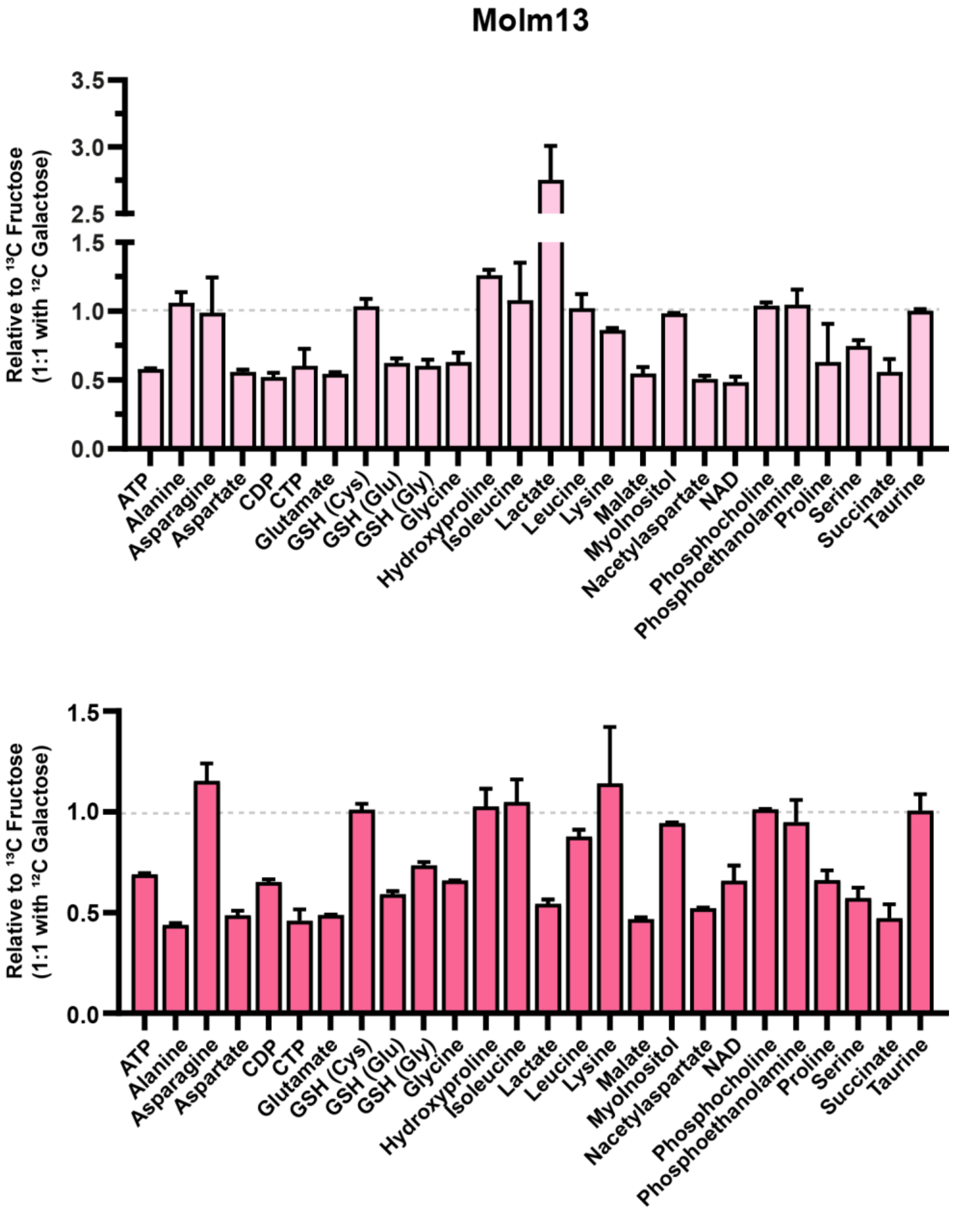

Supplementary Figure S3. Metabolic competition assay of fructose and galactose. Bar plots displaying the ^{13}C Intensity of a panel of metabolites labelled with ^{13}C galactose (mixed 1:1 with ^{12}C Fructose) in Molm13 and THP1 cells relative to those labelled with ^{13}C fructose (mixed 1:1 with ^{12}C Galactose.

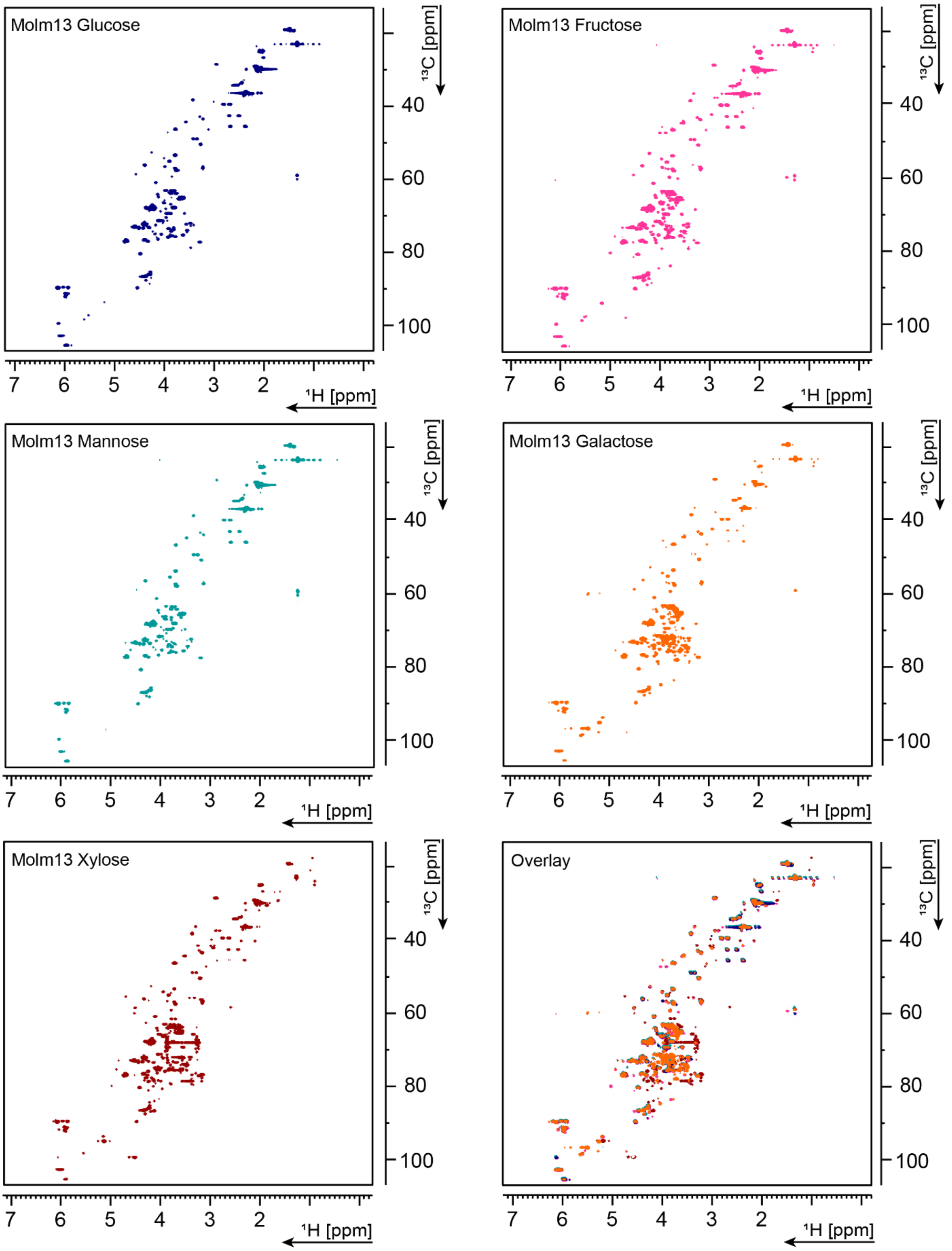

Supplementary Figure S4. ^{1}H-^{13}C 2D-HSQC NMR spectra of ^{13}C labelled sugars (glucose, fructose, mannose, galactose and xylose. An overlaid spectrum of all sugars is also shown.
